# Sphingosine-1-phosphate is a novel biomarker in sepsis severity

**DOI:** 10.1186/2197-425X-3-S1-A789

**Published:** 2015-10-01

**Authors:** A Nierhaus, MS Winkler, M Holzmann, E Mudersbach, A Bauer, L Robbe, C Zahrte, E Schwedhelm, G Daum, S Kluge, C Zoellner

**Affiliations:** University Medical Center Hamburg-Eppendorf, Department of Intensive Care Medicine, Hamburg, Germany; University Medical Center Hamburg-Eppendorf, Department of Anaesthesiology, Hamburg, Germany; University Medical Center Hamburg-Eppendorf, Institute of Clinical Pharmacology and Toxicology, Hamburg, Germany; University Heart Center, University Medical Center Hamburg-Eppendorf, Clinic and Polyclinic for Vascular Medicine, Hamburg, Germany

## Objectives

Sepsis is characterized by capillary leakage followed by organ dysfunction. Sphingosine-1-phosphate (S1P) is a signalling lipid that regulates endothelial permeability. Here we investigated whether serum S1P concentrations are associated with sepsis severity.

## Methods

Primary outcome variable was the serum S1P concentration quantified by mass spectrometry. Blood was drawn at day of enrollment. S1P was correlated with inflammatory markers and Sequential Organ Failure Assessment Score (SOFA) score for sepsis severity.

## Results

All three groups of patients had significantly (P < 0.001) lower serum S1P concentrations than controls (median 457.9 µg/L; interquartile range, IQR 379.8-562.3 µg/L). The lowest S1P concentrations was found in the septic shock group (149.1 µg/L; IQR, 105.1-163.2 µg/L) compared to patients with severe sepsis (234.7 µg/L; IQR, 185.4-342.6 µg/L) and sepsis (246.7 µg/L; IQR, 201.7-306.0 µg/L). S1P levels were positively correlated with high-density lipoprotein (HDL), and red blood cell count (RBC) and negatively correlated with: procalcitonin, interleukin-6, C-reactive protein and lactate. In a multivariate linear regression model S1P, HDL and RBC were significantly associated with SOFA score (P < 0.001). S1P concentration below 158.2 µg/l was the most potent indicator for diagnosing sepsis with shock compared to all other inflammatory markers. In a multivariate logistic regression model calculated for prediction of septic shock the Odds ratio for S1P was 0.97 (CI 95% 0.96-0.99) and the best predictor of shock (P < 0.01) among all parameters tested.

## Conclusions

S1P is an indicator of sepsis severity and superior to predict septic shock than currently established markers. Further prospective studies are needed to confirm the results in a larger cohort of non-septic patients and controls.

